# Gastric Trichobezoar: An Enduring Intrigue

**DOI:** 10.1155/2012/136963

**Published:** 2012-11-28

**Authors:** S. Mewa Kinoo, B. Singh

**Affiliations:** Department of Surgery, Nelson R Mandela School of Medicine, University of KwaZulu-Natal, 719 Umbilo Road, Congella 4013, South Africa

## Abstract

Trichobezoar is a rare condition that may pose a diagnostic challenge. Patients with this condition often have an underlying psychiatric illness, and history may not be easily forthcoming. The condition should be entertained especially in young females. Delay in diagnosis may lead to futile complications. We report a classic case of trichobezoar in terms of patient profile, presentation, and investigative findings.

## 1. Introduction

A bezoar is a mass of undigested material within the gastrointestinal tract. The term bezoar derives from the Arabic word *Badzehr*, which means antidote [1]. Bezoars were used as antidotes against plague, snake-bite, leprosy, and epilepsy by physicians from 12th to 18th century [2]. Trichobezoar is from the Greek word *trich* which means hair [3]. A trichobezoar is a mass of undigested hair within the gastrointestinal tract. Trichobezoars are often associated with trichotillomania (hair pulling), and trichophagia (hair swallowing). Trichotillomania may be unconsciously or unintentionally done and is part of the DSM IV psychiatric classification of impulse control disorders [4, 5]. In up to 18% of patients with trichotillomania, trichophagia occurs; one third of patients with trichophagia develop trichobezoars [6]. Trichobezoars most commonly occur in adolescent females [7]. The site of hair pulling is most commonly from the scalp, but can occur from the eyelashes, eyebrows, and pubic area [8].

## 2. Case Presentation

**Figure 1:**
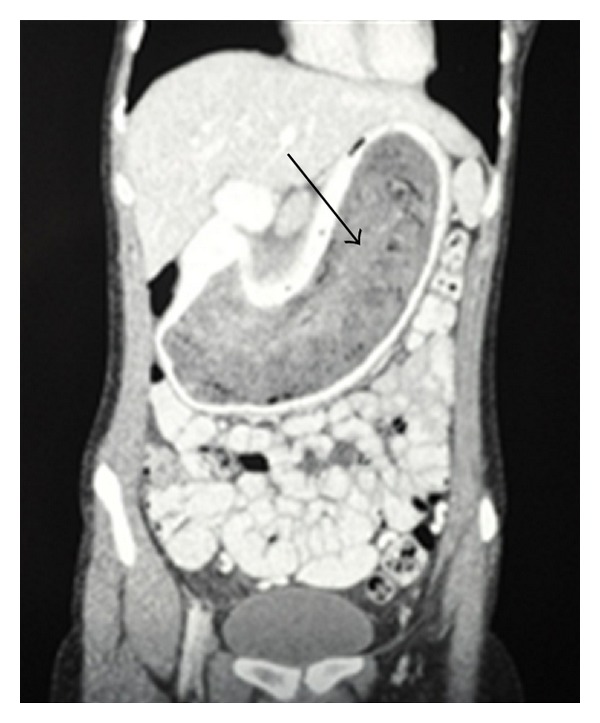
CT scan demonstrating trichobezoar (arrow) occupying the extent of the stomach.

**Figure 2:**
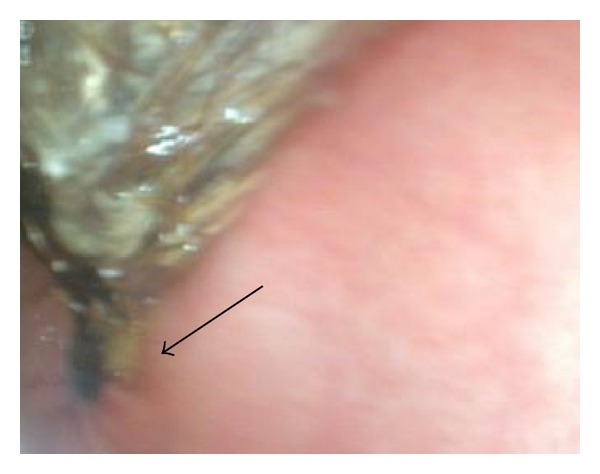
Endoscopy demonstrating trichobezoar extending through pylorus (arrow).

**Figure 3:**
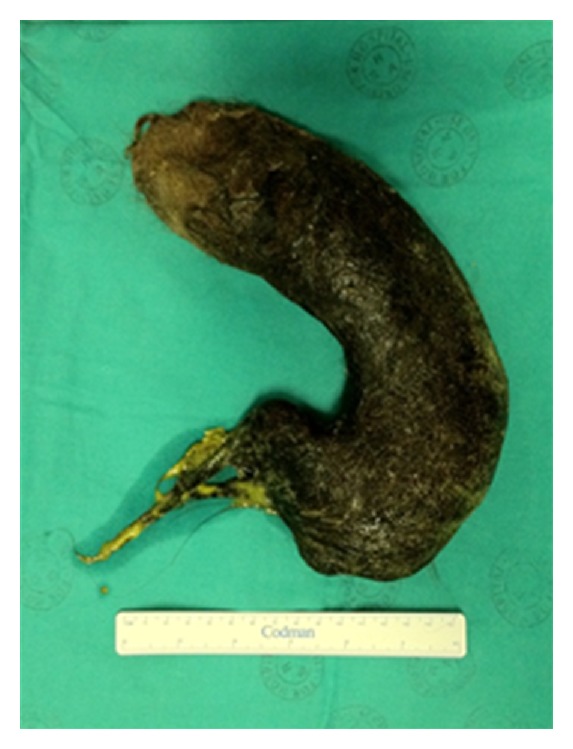
Gastric cast trichobezoar that was removed via gastrostomy.

A 16-year-old female was referred to our surgical services with a problem of epigastric pain associated with an epigastric mass. The patient resided in a local children's home due to poor social circumstances. On questioning, patient reported nonspecific pain of approximately 3 months duration with no dyspeptic symptoms; the patient reported no early satiety and no history of weight loss. Examination revealed a well looking girl, with a nondistended abdomen. Palpation of her abdomen revealed a large, firm mobile epigastric mass that was minimally tender. Ultrasound and Computed Tomography (CT) scan of the abdomen both confirmed the presence of a large gastric mass with internal air loculi involving the entire stomach with extention into the duodenum but not into the jejunum (Figure 1). At endoscopy a trichobezoar, involving almost the entire capacity of the stomach, extending from the distal oesophagus into the duodenum was noted (Figure 2). Prompted by these diagnostic findings, on further enquiry, the patient admitted to trichophagia. The patient was referred to the hospital psychiatrist, social worker, and psychologist for counselling. Due to the size of the trichobezoar and potential for complications, operative removal of the trichobezoar was undertaken successfully via a gastrostomy (Figure 3). The patient was discharged well on day 5 into the care of the psychologists.

## 3. Discussion

Trichobezoars commonly occur in adolescent females, often with an underlying psychiatric or social problem. Clinical presentation of these patients may be confusing as often they are not forthcoming with a history of trichophagia either due to embarrassment or the unintentional nature of the problem. Although this is a rare condition, numerous case reports and series have been reported as high mortality may follow complications associated with this condition. 

Trichobezoars in humans were first described from a post mortem by Swain in 1854 [9]. The postulated reason for formation in the stomach is that hair is undigestable and due to its smooth nature cannot be propulsed with peristalsis and over time forms a bezoar within the stomach. This bezoar can extend distally from the stomach into the caecum. Extention of the bezoar from the stomach into the jejunum or further on is referred to as “Rapunzel syndrome,” first described by Vaughan Jr. et al. in 1968 [10]. Rapunzel was a long haired girl in a German fairy tale by Grimm brothers. Bezoars can also be found distally in the gastrointestinal tract without continuity with the stomach bezoar due to breakage and distal propulsion. Trichobezoars continue to grow in size with continued ingestion of hair and this increases the risk of severe complications. The most common of these complications that have been reported over the years include gastric mucosal erosion, ulceration, and perforation of the stomach or the small intestine, gastric outlet obstruction, intussusception, obstructive jaundice, protein-losing enteropathy, pancreatitis, and death [11–16].

Presentation ranges from nonspecific abdominal or epigastric pain, to a range of complications as mentioned. Clinical examination often reveals a large mobile epigastric mass that may be indentable, the so-called Lamerton's sign [17]. Endoscopy is usually diagnostic. The hair appears black (despite the normal hair colour) due to denaturing of the hair protein by the acid. The most common diagnostic tool used in the literature is a CT scan, with a typical image showing a well-defined intraluminal ovoid heterogeneous mass with interspersed gas [18, 19]. 

Management options include endoscopic removal, laparoscopic removal, or via laparotomy. Gorter et al., in a retrospective review of 108 cases of trichobezoar, evaluated the available management options [20]; it was noted that whereas 5% of attempted endoscopic removals were successful, 75% of attempted laparoscopies were successful. However, laparotomy was 100% successful and thus favoured as their management of choice.

## 4. Conclusion

Trichobezoars should be considered as a differential diagnosis in a young female patient with a mobile epigastric mass. Diagnosis can be easily made with the use of CT scan and endoscopy. Management almost always requires surgical removal. It is emphasized that the majority of these patients have an underlying psychiatric or social disorder. A multidisciplinary approach is essential to prevent recurrence of the problem.
